# Hypersensitivity to Ticks and Lyme Disease Risk

**DOI:** 10.3201/eid1101.040303

**Published:** 2005-01

**Authors:** Georgine Burke, Stephen K. Wikel, Andrew Spielman, Sam R. Telford, Kathleen McKay, Peter J. Krause, the Tick-borne Infection Study Group

**Affiliations:** * Connecticut Children's Medical Center, Hartford, Connecticut, USA;; † University of Connecticut School of Medicine, Hartford, Connecticut, USA;; ‡ Harvard University, Cambridge, Massachusetts, USA;; § Tufts University School of Veterinary Medicine, North Grafton, Massachusetts, USA

**Keywords:** Lyme disease, anti-tick immune protection, hypersensitivity, research

## Abstract

Although residents of Lyme disease–endemic regions describe frequent exposure to ticks, Lyme disease develops in relatively few. To determine whether people who experience cutaneous hypersensitivity against tick bite have fewer episodes of Lyme disease than those who do not, we examined several factors that might restrict the incidence of Lyme disease among residents of Block Island, Rhode Island. Of 1,498 study participants, 27% (95% confidence interval [CI] 23%–31%) reported >1 tick bites, and 17% (95% CI 13%–21%) reported itch associated with tick bite in the previous year. *Borrelia burgdorferi* infected 23% (95% CI 20%–26%) of 135 nymphal *Ixodes scapularis* (*I. dammini*) ticks. The likelihood of Lyme disease infection decreased with >3 reports of tick-associated itch (odds ratio 0.14, 95% CI 0.94–0.03, p = 0.01). Prior exposure to uninfected vector ticks protects residents of disease-endemic sites from Lyme disease.

## Abstract 2

Persons who report frequent tick-associated itch are less likely to contract Lyme disease than those who do not.

Although many residents of Lyme disease–endemic regions describe frequent exposure to ticks, relatively few become infected by the causative spirochetal agent, *Borrelia burgdorferi* (*1**–**4*). This disparity reflects both a relative paucity of spirochetal infection in vector ticks and the limited number of people actually bitten by ticks (*5**–**6*). Other variables that might restrict Lyme disease incidence include prompt removal of attached ticks before the pathogen is transmitted and acquired immunity to the salivary proteins of these ticks, the spirochetal pathogen, or both (*7**–**11*). Repeated exposure to tick bites has been associated with developing cutaneous hypersensitivity, which results in persistent itch and local swelling at the site of tick attachment (*12**–**13*). Itching provides an early sign of tick bite and may facilitate removal of the attached tick before the pathogen can be transmitted. Additional inflammatory reaction to tick salivary proteins also may help prevent transmission (*10**–**11*). The epidemiologic relevance of host immunity to tick bite for preventing Lyme disease remains unknown.

Acquired immunity to vector ticks may limit the incidence of Lyme disease by protecting persons who have been previously exposed to bites of vector ticks. Accordingly, we determined whether cutaneous hypersensitivity against tick antigens increases with the frequency of tick exposure and whether such reactivity protects against Lyme disease. In particular, we determined whether residents of Block Island, Rhode Island, who experienced itching associated with attached ticks have fewer episodes of Lyme disease than those who report no episodes of itching associated with tick attachment.

## Methods

### Study Site and Sampling Procedures

Block Island is located 15 km from the New England mainland, and Lyme disease is highly endemic there. Beginning in 1991, we invited all residents to participate in a serosurvey twice yearly (during October and April). We sought to identify all cases of infection due to *B. burgdorferi* among serosurvey participants with the help of the staff of the Block Island Medical Center, the sole medical facility on the island, and by a dedicated research nurse (*3*). Borrelial infections were identified among members of the study cohort who visited the medical center for an acute tickborne illness or who seroconverted against borrelial antigen during the study period. Written informed consent was obtained from all adult study participants and from the parents or legal guardians of minors, in accordance with human experimentation guidelines approved by the institutional review boards at Connecticut Children's Medical Center and the Harvard School of Public Health.

### Data Collection

At the serosurvey visit, participants submitted a blood sample and responded to a questionnaire about tick bites, use of protective measures, exposure factors, and symptoms of tickborne illness during the previous year (Table A1). Specifically, they were asked if they had experienced a tick bite in the previous year and whether their tick bites produced itch. A medical history, physical examination, and specific Lyme disease laboratory tests were performed on all symptomatic participants at the time of suspected Lyme disease illness and 4–6 weeks later. A medical history was repeated at least every 3 months until the participants became asymptomatic. *Ixodes scapularis* ticks (*I. dammini*) were collected in May through October from 1991 through 2000 by flagging at diverse sites on the island. Ticks were analyzed for *B. burgdorferi* by polymerase chain reaction (PCR).

### PCR Assay for Spirochete DNA

Whole blood samples were analyzed and processed by personnel blinded to the clinical status of the donor, as previously described (*14*). DNA extraction was performed on blood from Lyme disease patients and on *Ixodes* ticks with a commercially available kit (IsoQuick Nucleic Acid Extraction Kit, ORCA Research, Bothell, WA) (*14**,**15*). A 294-bp portion of the *B. burgdorferi* OspA gene was targeted for amplification by using a previously described PCR protocol (*14**,**15*).

### Assays for Antispirochetal Antibody

Serologic evidence of exposure to the Lyme disease spirochete was detected by enzyme-linked immunosorbent assay (*16*). A reactive serum was defined as one with a positive reaction at a dilution >1:320. All borderline or reactive sera were further characterized by immunoblot (*16**,**17*). Specimens were considered positive if the immunoglobulin (Ig) G immunoblot contained >5 of the 10 most common *B. burgdorferi*–specific bands (*17*).

### Case Definition

To include both symptomatic and asymptomatic cases of Lyme disease, diagnosis of newly acquired *B. burgdorferi* infection during the course of the study required one of the following: 1) a physician diagnosis of erythema migrans consisting of an expanding, ringlike erythematous rash at least 5 cm in diameter; 2) influenzalike symptoms consistent with Lyme disease and laboratory evidence of recent infection; 3) seroconversion from an initial nonreactive serum to a subsequent reactive serum that contained anti-*B. burgdorferi* antibody. The influenzalike symptoms of Lyme disease include fever, chills, sweats, fatigue, headache, or myalgia. Laboratory evidence of recent infection included either amplification of *B. burgdorferi* DNA in blood by PCR, seroconversion, or a 4-fold rise in anti-*B. burgdorferi* antibody in paired acute-phase and convalescent-phase sera.

### Predicted and Observed Lyme Disease Rates

A simple model of Lyme disease transmission would calculate the rate of Lyme disease infection as the product of 2 main factors: the proportion of persons who report being bitten by deer ticks and the proportion of these ticks infected by *B. burgdorferi*. We calculated the yearly incidence of Lyme disease by determining the number of serosurvey participants who met the Lyme disease case definition each year in relation to the number of participants enrolled each year. By comparing the projected incidence using the Lyme disease transmission model to actual incidence of Lyme disease, we could assess the overall importance of factors missing from the basic model, such as the effects of inflammatory reactions against tick salivary proteins and acquired immunity to the spirochetal pathogen.

### Statistical Analysis

All statistical calculations were performed with JMP 5.1 (SAS Institute, Cary, NC). To estimate the study sample frequency of tick bite and tick-associated itch, we averaged the individual reports of tick bite and tick-associated itch among study participants each year. To determine yearly Lyme disease incidence, we compared the number of new cases of Lyme disease each year to the total number of study participants who had enrolled in the study up to that time. To create 10-year individual measures, reports of tick bite and itch were summed across all visits for each participant. The results were analyzed with descriptive statistics (mean, proportion, and confidence intervals with 5% error). Bivariate logistic regression was used to estimate probability of itch for increasing tick bites. The relative contribution of risk factors to the acquisition of Lyme disease was evaluated from multiple logistic regression models to calculate odds ratios with associated confidence limits. The predicted probability of acquiring Lyme disease was estimated for significant risk factors.

## Results

A total of 1,669 residents of Block Island, most of the island population, enrolled in our study from 1991 to 2000. We excluded those participants who did not report spending at least 1 month on the island during May through October, which resulted in a sample of 1,490. The mean age of the sample was 43 years (95% CI 42.2–44.1) and approximately half (51%) were female.

**Figure 1:**
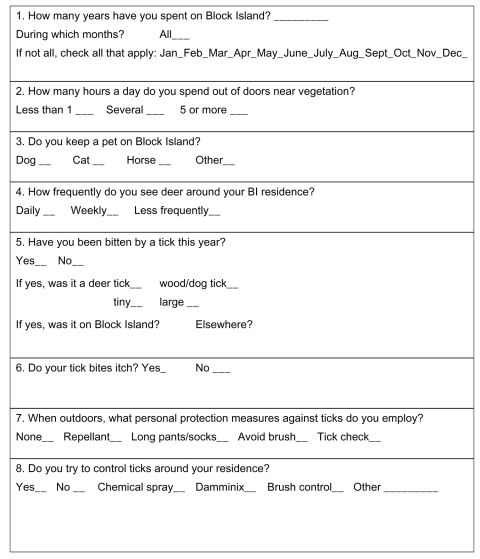
Reported tick bite and itch by serosurvey year on Block Island, Rhode Island, 1991–2000.

We determined how frequently the 1,490 persons in our sample recalled a deer tick bite. Each year an average of 27% (95% CI 23%–31%) of the study participants reported >1 tick bite during the prior year (range 20%–37%). An average of 17% (95% CI 13%–21%) of study participants reported itch associated with tick bite (Figure 1).

We then determined the prevalence of *B. burgdorferi* infection in nymphal deer ticks that contained *B. burgdorferi* DNA and that were swept from vegetation on Block Island throughout the course of this study. Of 135 such ticks tested, 23% (95% CI 20%–26%) contained amplifiable *B. burgdorferi* DNA. We therefore predicted that the maximum yearly incidence of Lyme disease would be 6.2% (95% CI 4.6%–8.1%) (27% with tick bite x 23% of ticks infected).

We next calculated yearly incidence rate of new Lyme disease cases among study participants from 1991 to 2000. The average incidence was 1.74% (95% CI 1.1%–2.4%) (Table). Thus, the predicted incidence of Lyme disease using the basic model (95% CI 4.6%–8.1%) was 3%–6% greater than the observed incidence. This difference would not be expected due to chance.

**Table:** Yearly incidence of Lyme disease among Block Island study cohort, 1991–2000*

Year	No. participants	No. (%) with first episode of Lyme disease
1991	731	25 (3.4)
1992	824	27 (3.3)
1993	933	17 (1.8)
1994	1,038	23 (2.2)
1995	1114	15 (1.4)
1996	1,169	15 (1.3)
1997	1,246	5 (0.4)
1998	1,325	18 (1.4)
1999	1,432	14 (1.0)
2000	1,490	19 (1.3)

*10-year average = 1.74 (95% confidence interval 1.1–2.4).

We next determined whether increasing exposure to ticks increases the probability of tick-associated itch. This analysis was limited to participants whose sera displayed no evidence of tickborne illness before study entry, who had >1 serosurvey visit, and who were >2 years of age. Among these 610 participants, 52% reported at least 1 tick bite (mean 2.2, 95% CI 2.0–2.4), and 32% reported itch with any tick bite. The probability of itch doubled as the number of reported tick bites increased from 1 to 2 (21% to 46%, respectively) and doubled again from 2 to 4 reported bites (46% to 97%, respectively; linear trend p < 0.001).

**Figure 2:**
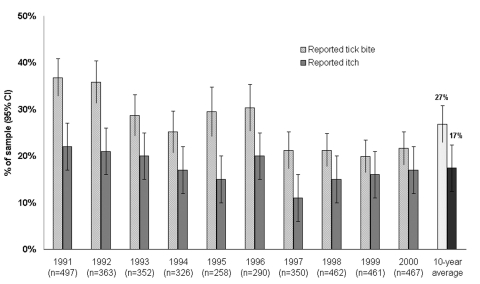
Risk of acquiring Lyme disease according to reports of tick bite–associated itch.

Finally, we tested the hypothesis that tick-associated itching is associated with decreased risk of Lyme disease. The acquisition of Lyme disease increased from 15% to 25% to 31% among participants who reported no itch, 1 episode of tick-associated itch, and 2 reports of itch, respectively. In contrast, the frequency of Lyme disease decreased to 13% in participants who reported 3 episodes of tick-associated itch and 10% in those with >4 such reports. We used a multiple logistic regression model to estimate the likelihood of acquiring Lyme disease for participants reporting none to 1, 2, and >3 reports of itch, controlling for number of study visits and reports of tick bite. Consistent with the bivariate analysis, the risk of acquiring Lyme disease was higher for 1 report of itch (OR 2.7, 95% CI 0.4–2.3), and decreased among those who reported itch at >3 study visits (range 3–12, OR 0.18, 95% CI 0.05–0.5) (Figure 2). Confining the analysis to only those who had Lyme disease illness by omitting participants whose evidence of Lyme disease was by seroconversion alone did not alter the inverse relationship between itch and developing Lyme disease. Persons who consistently report itching in association with tick bites are less likely to experience an episode of Lyme disease than do those who fail to react against tick bite.

## Discussion

These observations suggest that residents of disease-endemic sites who experience persistent tick-associated itch are less likely to develop Lyme disease than are those who do not experience this reaction. Itch was reported at the site of tick bite, and the frequency of itch increases as the number of reported tick bites increase, which strongly suggests that tick-associated itch is associated with an acquired cutaneous hypersensitivity response. Such a relationship has definitively been established in the case of *I. ricinis*, the European analog of the North American deer tick. After having been bitten by these ticks repeatedly, persons experience both immediate and delayed cutaneous hypersensitivity reactions. They express tick-specific IgE antibodies, as well as dermal and perivascular infiltrates of CD8+ T lymphocytes and Langerhans' cells (*13*). Additional studies of other tick species also suggest that tick-associated itch is mediated by tick-specific antibody or cellular infiltration (*12**,**18**,**19*). People in our study who reported a single episode of tick-associated itch were more likely to acquire Lyme disease than those who did not report itch, probably because tick-associated itch is a marker for tick exposure. In contrast, persons who had repeated tick-associated itch were protected from developing Lyme disease. We are confident, therefore, that the people in our study who described repeated episodes of tick-associated itching were experiencing cutaneous hypersensitivity and that this immune reaction protected them from acquiring Lyme disease.

The antitick immune response that protects people from acquiring Lyme disease might act through any of several effector mechanisms. A heightened awareness by a person of an attached tick would result in the removal of the potentially infecting tick before pathogen transmission could occur. Because vector ticks must remain attached for at least 2 days before spirochetes are transmitted, prompt tick removal should prevent spirochetal infection (*8**,**20*). Vector ticks generally are removed by persons within such a time period (*21**–**23*). Alternatively, immunity against tick salivary antigens might interfere with pathogen transmission independent of early host recognition and removal of ticks. Indeed, preexposure of mice and guinea pigs to uninfected ticks prevents Lyme disease after challenge by spirochete-infected ticks using infestation conditions that prevent removal of feeding ticks by host grooming (*24**,**25*). Ticks feeding on such immune hosts detach sooner and retain less host material than do ticks feeding on nonimmune hosts. Although natural hosts, such as *Peromyscus leucopus*, do not develop robust antitick immunity, inbred strains of mice and guinea pigs become immune to the salivary antigens secreted by feeding ticks (*10**,**26**,**27*). This immunity results in reduced volume of engorgement, abnormal blood meal composition, prolonged feeding, and frequently death of the tick (*10**,**27*). Antitick immunity may specifically neutralize some components of tick saliva that ensure successful feeding and facilitate pathogen transmission, such as vasodilators, anticoagulants, and immunosuppressors (*10**,**11**,**28*). Prevention of *B. burgdorferi* transmission in guinea pigs is associated with antivector antibody (*25*). Persons who experience frequent deer tick bites produce an array of specific antitick antibodies (*29**–**31*). Cell-mediated immune factors against tick-derived antigen might similarly play a role in developing cutaneous hypersensitivity and protect against infection (*13**,**19*). A strong delayed cutaneous hypersensitivity response to sandfly bites in persons has been correlated with reduced transmission of leishmanial parasites (*32**,**33*). An array of antitick immune reactions may prevent Lyme disease and other tickborne diseases.

Our analysis incorporates several potentially confounding assumptions. Although our estimate of the prevalence of spirochetal infection in vector ticks is based on a small sample of ticks, this rate is consistent with that derived in other parts of southern New England at approximately the same time (*5**,**6*). Although our study participants live on Block Island where deer ticks predominate, some of our participants may have been bitten by other kinds of ticks or been bitten by ticks off the island. While approximately a quarter of our participants reported a tick bite, previous studies have shown that persons are often unaware that they have been bitten. From a third to three fourths of patients with Lyme disease cannot recall having been bitten by a tick (*6**,**34**–**36*). Assuming that a similar percentage of our study participants were unaware of a previous tick bite, the tick bite prevalence on Block Island would have varied from half to all of our participants. A tick bite prevalence of 71% was reported at another site where Lyme disease is highly endemic (*37*). Any underestimate of the frequency of tick attachment would increase the likelihood that other factors, including antitick immunity, help limit the incidence of Lyme disease. Finally, our predicted Lyme disease incidence assumes that people are bitten only once in any year. An increase in yearly tick bite frequency among persons would increase the likelihood that the tick bite is from an infected tick, thus similarly increasing the difference between predicted and observed incidence rates. We believe that none of these assumptions is notably confounding.

Several factors help prevent Lyme disease in persons who live in disease-endemic regions. These factors include the paucity of ticks that are infected with the Lyme spirochete, the limited number of persons who are bitten by ticks, acquired immunity against the spirochetal pathogen, and the immune reaction to ticks that develops in the course of tick attachment. Persons who express an immune reaction against the vector tick appear to acquire Lyme disease less frequently than do those who experience no such immune reaction. The protective effect of the immune response to tick salivary protein against the agent of Lyme disease in persons suggests that a tick protein-based vaccine might be developed that would protect against infection by the agent of Lyme disease and possibly other tickborne infections.

## Appendix

**Table A1:** Tick exposure history in standardized serosurvey questionnaire

1. How many years have you spent on Block Island? _________
During which months? All___ If not all, check all that apply: Jan_Feb_Mar_Apr_May_June_July_Aug_Sept_Oct_Nov_Dec_
2. How many hours a day do you spend out of doors near vegetation? Less than 1 ___Several ___ 5 or more ___
3. Do you keep a pet on Block Island? Dog __ Cat __ Horse __ Other__
4. How frequently do you see deer around your BI residence? Daily __ Weekly__ Less frequently__
5. Have you been bitten by a tick this year? Yes__ No__
If yes, was it a deer tick__ wood/dog tick__ tiny__ large __
If yes, was it on Block Island? Elsewhere?
6. Do your tick bites itch? Yes_ No ___
7. When outdoors, what personal protection measures against ticks do you employ? None__ Repellant__ Long pants/socks__ Avoid brush__ Tick check__
8. Do you try to control ticks around your residence? Yes__ No __ Chemical spray__ Damminix__ Brush control__ Other ________
